# The Association Between Callous–Unemotional Traits, Externalizing Problems, and Gender in Predicting Cognitive and Affective Morality Judgments in Adolescence

**DOI:** 10.1007/s10964-016-0527-x

**Published:** 2016-06-22

**Authors:** Iro Fragkaki, Maaike Cima, Cor Meesters

**Affiliations:** 1 Behavioural Science Institute, Radboud University Nijmegen, Montessorilaan 3, 6525 HE Nijmegen, The Netherlands; 2 Department of Clinical Psychological Science, Maastricht University, Minderbroedersberg 4-6, 6211 LK Maastricht, The Netherlands

**Keywords:** Callous–unemotional traits, Externalizing problems, Gender, Morality judgments

## Abstract

Morality deficits have been linked to callous–unemotional traits and externalizing problems in response to moral dilemmas, but these associations are still obscure in response to antisocial acts in adolescence. Limited evidence on young boys suggested that callous–unemotional traits and externalizing problems were associated with affective but not cognitive morality judgments. The present study investigated these associations in a community sample of 277 adolescents (*M*_*age*_ = 15.35, 64 % females). Adolescents with high callous–unemotional traits showed deficits in affective but not cognitive morality, indicating that they can identify the appropriate moral emotions in others, but experience deviant moral emotions when imagining themselves committing antisocial acts. Externalizing problems and male gender were also strongly related to deficits in affective morality, but they had smaller associations with deficits in cognitive morality too. Implications for treatment and the justice system are discussed.

## Introduction

Callous–unemotional traits and externalizing problems are related to aggression and delinquency that may lead to criminal behavior in the long run with detrimental personal consequences and a substantial societal burden (Colman et al. 2009; Fergusson et al. 2005; Frick et al. 2014; Kimonis et al. 2014; Odgers et al. 2007, 2008). Externalizing problems include aggressive and delinquent behavior and they are sometimes comorbid with callous–unemotional traits leading to more severe antisocial behavior (Frick and White 2008; Frick et al. 2014). However, adolescents who present only callous–unemotional traits have specific characteristics such as lack of empathy, shame, or guilt and shallow emotion (Frick and White 2008; Frick et al. 2014). A large body of research has revealed that externalizing problems as well as callous–unemotional traits are associated with moral deficits (Malti and Krettenauer 2013; Stams et al. 2006). This line of research has primarily focused on moral development and moral emotions in response to moral dilemmas. Moral dilemmas describe situations in which people have to decide whether to break a moral rule for personal gain (e.g., find a wallet and not returning it to the owner to keep the money), help others in need, and sacrifice one person to save many (eg. trolley dilemma; Foot 1967). However, morality is a multidimensional construct that influences a broad spectrum of behaviors and decisions in our life that extend beyond theoretical moral dilemmas. For instance, aggressive and delinquent acts include a moral component and they are usually perceived as morally unacceptable actions that should elicit negative moral emotions (e.g., guilt). A critical question that has not been sufficiently explored yet is how adolescents with externalizing problems and/or callous–unemotional traits who are at risk of delinquency perceive antisocial acts from a moral perspective and what moral emotions they attribute to these acts.

Morality is the ability to discriminate between right and wrong based on the rules of ethics and societal norms and behave accordingly (Koops et al. 2010; Smetana et al. 2000). Moral development is a process that starts in early childhood and continues into adolescence and adulthood. Children at age 4–5 perceive several behaviors as immoral, but they attribute positive emotions in a person who commits an immoral act to achieve a desired object, a phenomenon called happy-victimizer response (Krettenauer et al. 2008). This response decreases over the course of development and positive feelings after a moral decision increase in adolescence (Krettenauer et al. 2014). Furthermore, morality has a cognitive and an affective component. Kohlberg (1984) identified three levels of moral development that develop over time, the preconventional, the conventional, and the postconventional. According to this approach, at the preconventional level rules are external to the self and imposed by authority figures, at the conventional level they become integrated to the self, and at the postconventional level the rules are differentiated from the self and the moral values become self-chosen principles (Colby and Kohlberg 1987). These moral stages constitute the core of cognitive morality. Individuals acquire the moral reasoning to distinguish right from wrong and behave accordingly, understand what another person feels under morally challenging situations and identify the related moral emotions in others (like shame, guilt, and empathy) (Koops et al. 2010; Smetana et al. 2000). Affective morality includes the personal moral emotions associated with moral situations or moral dilemmas. Moral emotions include positive emotions (happiness, excitement) and negative emotions (guilt, shame, fear, sadness) and they can be measured by asking the participants how they would feel in morally challenging situations. In addition, affective morality involves situations where the moral emotions direct our behavior without a moral reasoning process, which means that our behavior is solely determined by our associated moral emotions (Koops et al. 2010; Smetana et al. 2000).

With respect to cognitive morality, numerous previous studies have revealed associations between cognitive morality deficits and externalizing problems or callous–unemotional traits. A meta-analysis on moral development revealed that juvenile delinquents showed a lower stage of moral development compared to non-delinquent adolescents and the effect sizes were larger for males and for those with callous–unemotional traits (Stams et al. 2006). Additionally, male adult psychopaths and boys with callous–unemotional traits fail to make the distinction between moral and conventional transgressions under modified rule conditions (Blair 1995, 1997; Blair et al. 2001; Dolan and Fullam 2010). These findings indicate that they perceive transgressions with negative consequences for the rights and welfare of others (moral) as equally forbidden as transgressions that include violations of the behavioral societal rules but are not forbidden by law (conventional) when the action is permissible by an authority figure (e.g., teacher). In contrast, other studies found no deficits in cognitive morality in male adult psychopathic offenders as they made the same moral judgments in moral dilemmas as non-psychopathic offenders and healthy controls, arguing that personal moral actions are less permissible than impersonal moral actions (Cima et al. 2010). The authors suggested that psychopaths seem to distinguish between right and wrong based on societal moral norms but fail to behave accordingly. A meta-analysis found an association between deviant moral emotions identified in others and aggressive behavior in children and adolescents (Malti and Krettenauer 2013). In addition, there were no gender differences in moral emotions identified in others. However, the role of callous–unemotional traits was not investigated and the included studies examined moral emotion attributions in response to moral dilemmas and not antisocial acts. Overall, the evidence suggests that externalizing problems are related to deficits in cognitive morality but the results on callous–unemotional traits are inconsistent. In addition, evidence on moral development are limited in males, raising questions about potential gender differences, whereas moral emotions identified in others in response to moral dilemmas do not seem to differ between males and females.

With respect to affective morality, the aforementioned meta-analysis by Malti and Krettenauer (2013) showed that externalizing problems were also related to self-attributed moral emotions (affective morality) and the effect sizes were larger than for the moral emotions identified in others (cognitive morality). Similar to cognitive morality, gender did not moderate the relationship between affective morality and externalizing problems. However, most of the studies measured moral emotions by asking the participants how bad or good they would feel if they commit an immoral act without specifying other moral emotions (e.g., shame, guilt, fear, excitement) and the vignettes included moral dilemmas and not antisocial acts. A limited number of studies have examined affective morality in response to aggressive acts. A study by Arsenio et al. (2004) examined affective morality judgments of happiness, sadness, anger, and fear in response to aggressive and nonaggressive events in adolescents with and without disruptive disorders at the age of 16. The adolescents with disruptive disorders reported lower scores in all moral emotions in response to nonaggressive situations and increased happiness in response to aggressive situations compared to healthy adolescents. However, this study focused on situations of proactive aggression and the relationship with callous–unemotional traits was not investigated.

A recent study on a community sample of boys aged 8–12 examined whether boys with externalizing problems and/or callous–unemotional traits differ from boys without these characteristics in cognitive and affective morality in response to antisocial acts measured by the Affective Morality Index (AMI; Cimbora and McIntosh 2003; Feilhauer et al. 2013). The AMI consists of ten short vignettes that display boys committing antisocial acts and the subjects are asked to identify the moral emotions of the protagonist (cognitive morality) and report how they would feel if they have committed the same act (affective morality) (Feilhauer et al. 2013). The findings revealed that callous–unemotional traits and externalizing problems were not associated with cognitive morality. In contrast, significant associations with affective morality were revealed. Boys with high callous–unemotional traits expressed higher feelings of happiness and excitement, lower feelings of guilt, and higher likelihood of committing a similar antisocial act (recidivism) and boys with high externalizing problems reported increased feelings of happiness when imagining themselves committing the antisocial acts. Moreover, there was an interaction between callous–unemotional traits and externalizing problems, indicating that boys with high callous–unemotional traits and externalizing problems expressed the highest levels of happiness and increased recidivism when imagining committing similar antisocial acts. Unfortunately, this study included only boys and thus gender differences were not explored. Considering that callous–unemotional traits and externalizing problems are higher in males both in community and clinical samples (Archer 2004; Bongers et al. 2004; Broidy et al. 2003; Chun and Mobley 2010; Cook et al. 2015; Essau et al. 2006; Euler et al. 2015; Meier et al. 2008; Stams et al. 2006; Urben et al. 2015), further research is needed to investigate potential gender differences and interactions in morality judgments of antisocial acts. Overall, the findings supported the notion that individuals with callous–unemotional traits and combined externalizing problems can distinguish between right and wrong and identify the related moral emotions in others (cognitive morality), but they experience more positive emotions and less negative emotions when imagining committing an antisocial act themselves, indicative of deficits in affective morality. This notion is in line with other scientific evidence demonstrating that callous–unemotional traits are consistently associated with deficits in affective empathy but not in cognitive empathy (Frick et al. 2014). Adolescents with high callous–unemotional traits can understand the perspective of others and identify their emotional state, but they have difficulties in sharing and responding compassionately to others’ emotions. Relatedly, it can be argued that they can discriminate between right and wrong and understand the feelings of the victims but they cannot empathize with them and instead they even experience positive emotions.

Taken together, although the existing studies have provided insight into the association between morality and externalizing problems as well as callous–unemotional traits, five important limitations should be mentioned. First, the majority of the studies examined moral emotions in response only to moral dilemmas and not to antisocial acts. Second, previous studies have primarily examined moral emotions by simply asking the participants how good or bad they would feel if they commit an immoral act. This measurement is limited and does not cover a broad range of other moral emotions, such as anger, guilt, shame, fear, or excitement. Third, the research on callous–unemotional traits and cognitive morality has yielded inconsistent results and the studies on affective morality and callous–unemotional traits are scarce. Fourth, there is a lack of research on the interaction between externalizing problems and callous–unemotional traits in cognitive and affective morality judgments in response to antisocial acts. To our knowledge, only one study examined this research question and it was conducted with children and not adolescents. Fifth, there is a lack of research on gender differences on morality judgments in response to antisocial acts.

## The Current Study

The aim of the present study was to address these issues and extend previous research on morality by investigating cognitive and affective morality in response to antisocial acts and their associations with callous–unemotional traits and externalizing problems in adolescence. Although the study by Feilhauer et al. (2013) provided a useful insight, it included only boys and was performed in a group of young children who had not reached adolescence yet. It therefore remains unknown whether the observed associations between callous–unemotional traits, externalizing problems and cognitive or affective morality are gender-specific and how they relate to adolescence. To fill this gap, we examined the associations between callous–unemotional traits, externalizing problems and cognitive or affective morality judgments covering a broad spectrum of moral emotions (anger, happiness, guilt, excitement, fear) in a large community sample of adolescents including both males and females. Our objective was to determine whether the main effects and interactions found in children (Feilhauer et al. 2013) were also present in adolescence and whether there were gender differences and interactions when externalizing problems are increased and callous–unemotional traits reach their peak at age 15–16 (Essau et al. 2006; Van Lier et al. 2007). Based on previous evidence, we hypothesized that (1) callous–unemotional traits and externalizing problems would be related to deficits in affective morality but not in cognitive morality, (2) adolescents with combined callous–unemotional traits and externalizing problems would present more deviant moral emotions in affective morality, and (3) we explored potential gender differences and we expected an interaction between gender and callous–unemotional traits or externalizing problems. Based on previous evidence indicating an association between male gender and higher callous–unemotional traits or externalizing problems and our hypothesized effect of these factors on affective morality, we expected that boys high callous–unemotional traits or externalizing problems would present the most pronounced deficits in affective morality.

## Methods

### Participants

The study included a community sample of 277 adolescents (99 boys, 178 girls) without any chronic illness at the time of the study and aged from 12 to 18 (*M* = 15.35, *SD* = 1.16). They were recruited from three public schools in Belgium (*n* = 201 students) and one school in the Netherlands (*n* = 76 students) where students were aged from 12 to 18. Not all of the students were approached due to particular school activities at the time of the study, such as holiday camp or exams. The available students and their parents were informed about the study and invited to participate. The response rate was 65.3 %. Written informed consent was obtained from all individual participants included in the study and their parents. The majority of the adolescents were living with both their parents (84.8 %), 13.4 % were living in a single-parent family, 1.1 % were living with foster parents or caregivers, and 0.4 % were living alone. From the total sample, 21.3 % (*n* = 58) had contact with the police (30 boys, 28 girls). The offenses were mostly misdemeanors, specifically traffic violations (*n* = 12), vandalism (*n* = 7), theft (*n* = 5), truancy (*n* = 1), mistreatment (*n* = 1), other (*n* = 28), and multiple reasons (*n* = 4). Seventy-one percent of the participants followed a high level track in school, 20 % followed a moderate level track, and 9 % followed a low level track.

### Procedure

The present study was conducted at the schools during school hours. The participants were asked to complete a battery of questionnaires and the duration of the administration was 30–40 min. The experimenter was present to explain the procedure and provide further clarifications. To avoid order effects, the questionnaires were counterbalanced. Participation in the study was completely voluntary and the participants were allowed to terminate their participation at any time. The compensation for participation was 20 vouchers of 15 euro that were raffled among the participants. The study was approved by the Ethical Committee of the faculty of Psychology of the Maastricht University.

### Instruments

#### Cognitive and Affective Morality Judgments

The Affective Morality Index (AMI; Cimbora and McIntosh 2003) was used to assess cognitive and affective morality. It consists of ten short vignettes that describe boys committing antisocial acts relevant to youth. After each story, the participants are asked to indicate how angry, happy, excited, guilty, afraid, and an “other” emotion they believe the protagonist would feel after committing the respective antisocial act on 4-point Likert scale (1 = *not at all*, 4 = *a lot*). In addition, they are asked how likely it is for the protagonist to commit the same antisocial act again (recidivism) on a 4-point Likert scale (1 = *definitely not*, 4 = *definitely yes*). These questions measure cognitive morality (AMI-OTHER) as the participants are asked to identify the moral emotions of the protagonist. To measure affective morality (AMI-SELF) the participants are asked how angry, happy, excited, guilty, afraid, and an “other” emotion they would feel themselves after committing the same antisocial acts and how likely it would be to commit the same act again on the same 4-point Likert scales, respectively. The antisocial acts displayed in the vignettes include stealing a CD from a store, start a serious fight, kick a dog to make him stop barking, swearing at the teacher, and change a bad grade in the teacher’s notebook. For each emotion, a proportion score is calculated by summing the scores for all ten vignettes and dividing it by the sum of all the emotion scores combined. The use of proportion scores controls for individual differences in the total emotional arousal (Cimbora and McIntosh 2003). Higher scores indicate higher levels of each emotion. The Cronbach’s α for the AMI-OTHER scores (cognitive morality) in this study were: .72 for anger, .70 for happiness, .78 for guilt, .74 for excitement, .80 for fear, and .81 for recidivism. The Cronbach’s α for the AMI-SELF scores (affective morality) in this study were: .84 for anger, .71 for happiness, .81 for guilt, .83 for excitement, .87 for fear, and .77 for recidivism.

#### Callous–Unemotional Traits

Callous–unemotional traits were assessed with the Inventory of Callous–Unemotional traits—Youth Version (ICU; Frick 2003), which is suitable for adolescents aged 13–17. It consists of 24 items and it has three subscales: callousness, uncaring, and unemotional, although other studies have suggested that five factors might be more appropriate for samples with offenders (Feilhauer et al. 2012). The total score was used in this study as an index of callous–unemotional traits. The items are rated on a 4-point Likert scale (0 = *not at all true*, 3 = *definitely true*). The ICU includes statements such as “I do not care who I hurt to get what I want” and “I seem very cold and uncaring to others”. The ICU is widely used in samples of healthy adolescents, juvenile delinquents and offenders in several countries and it has good internal consistency, and good construct, convergent and discriminant validity (Essau et al. 2006; Fanti et al. 2013; Feilhauer et al. 2012; Kimonis et al. 2008, 2014; Roose et al. 2010). In this study the Cronbach’s α of the total scale was .76.

#### Externalizing Problems

The Youth Self-Report (YSR; Achenbach 1991) measures externalizing and internalizing psychopathological problems. In this study, we used only the 20 items referring to externalizing problems. Participants are asked to indicate the frequency of several behavioral symptoms on a 3-point Likert scale (0 = *not true*, 2 = *very true/often true*). Examples of the YSR items are “I destroy things belonging to others” (aggression) and “I set fires” (delinquency). The YSR is a widely used instrument for psychopathological symptoms in childhood and adolescence with well-established psychometric properties (Ebesutani et al. 2011). In this study, the Cronbach’s α was .81.

## Results

### Descriptive Statistics

There were no gender differences in age, *t*(275) = 1.081, *p* = .281, or externalizing problems, *t*(275) = −.791, *p* = .430. There were significant gender differences in callous–unemotional traits, *t*(275) = −4.287, *p* < .001, indicating that boys had significantly higher callous–unemotional traits than girls. Boys were also more likely to have contact with the police than girls, χ^2^(1, *N* = 277) = 8.161, *p* = .004, OR = 2.329, 95 % CI [1.293, 4.196]. With respect to cognitive morality judgments, boys perceived the protagonist as more excited and happy as well as less guilty and angry than girls (all *p*s < .05). Regarding affective morality judgments, boys reported increased feelings of happiness, excitement and perceived an increased likelihood of recidivism, as well as decreased feelings of guilt and fear when imagining themselves committing similar antisocial acts (all *p*s < .001). Table 1 presents all the means and standard deviations for the total sample and for each gender separately. Adolescents from Belgian schools had significantly higher externalizing problems (*M* = 7.58, *SD* = 4.59) than adolescents from Dutch schools (*M* = 5.01, *SD* = 3.49), *t*(275) = 4.418, *p* < .001, and more offenses, χ^2^(1, *Ν* = 277) = 5.589, *p* = .018, OR = .406, 95 % CI [.189, .872]. Adolescents from Belgian schools were significantly older (*M* = 15.72, *SD* = 1.09) than adolescents from Dutch schools (*M* = 14.38, *SD* = 0.69), *t*(212) = 12.11, *p* < .001. Educational level was not related to the total scores of externalizing problems, *F*(2, 274) = .991, *p* = .373, or callous–unemotional traits, *F*(2, 274) = 1.509, *p* = .223.

**Table 1:** Means and standard deviations for the total sample and each gender separately

	Total	Girls	Boys	*t*
	*M (SD)*	*M (SD)*	*M (SD)*	
*ICU*				
CU traits	22.90 (7.29)	21.54 (6.54)	25.34 (7.92)	−4.29***
*YSR*				
Externalizing problems	6.88 (4.46)	6.71 (4.40)	7.16 (4.58)	−.79
*AMI*-*OTHER*				
Anger	0.18 (0.03)	0.18 (0.03)	0.17 (0.03)	2.46*
Happy	0.17 (0.04)	0.16 (0.04)	0.18 (0.03)	−3.75***
Guilt	0.24 (0.05)	0.25 (0.04)	0.23 (0.05)	2.45*
Excitement	0.19 (0.05)	0.18 (0.05)	0.20 (0.04)	−2.18*
Fear	0.22 (0.04)	0.22 (0.04)	0.21 (0.04)	1.45
Recidivism	2.76 (0.69)	2.75 (0.59)	2.77 (0.84)	−1.19
*AMI*-*SELF*				
Anger	0.18 (0.04)	0.18 (0.04)	0.18 (0.04)	.51
Happy	0.13 (0.04)	0.12 (0.03)	0.15 (0.04)	−6.76***
Guilt	0.30 (0.04)	0.31 (0.04)	0.28 (0.50)	4.10***
Excitement	0.14 (0.04)	0.13 (0.03)	0.16 (0.04)	−6.30***
Fear	0.25 (0.04)	0.26 (0.04)	0.22 (0.04)	7.22***
Recidivism	1.34 (0.64)	1.29 (0.56)	1.43 (0.76)	−3.57

*ICU* Inventory of Callous–Unemotional traits; *CU traits* Callous–unemotional traits; *YSR* Youth Self-Report; *AMI* Affective Morality Index; *AMI-OTHER* Cognitive Morality Index; *AMI-SELF* Affective Morality Index

* *p* < .05; ** *p* < .01; *** *p* < .001

### Correlation Analysis

Table 2 presents the correlations between the AMI scales of cognitive and affective morality, callous–unemotional traits, and externalizing problems. There were significant positive correlations between positive emotions (happiness, excitement) and significant negative correlations between positive (happiness, excitement) and negative emotions (guilt, fear). Recidivism was positively correlated with happiness and excitement and negatively correlated with guilt and fear. Callous–unemotional traits were positively correlated with externalizing problems, suggesting that adolescents with externalizing problems had also increased callous–unemotional traits. With respect to cognitive morality, high callous–unemotional traits were related to increased feelings of happiness and excitement, and decreased feelings of anger and guilt identified in the protagonist. Externalizing problems were significantly associated with increased feelings of excitement, perceived likelihood of recidivism, and decreased feelings of anger and guilt. With respect to affective morality, high callous–unemotional traits were significantly associated with increased feelings of happiness and excitement, increased perceived likelihood of recidivism, and decreased feelings of guilt and fear. Externalizing problems were significantly correlated with increased feelings of happiness and excitement, increased perceived likelihood of recidivism, and decreased feelings of anger, guilt, and fear.

**Table 2:** Correlations among callous–unemotional traits, externalizing problems, and morality judgments

	ICU	YSR	AMI-OTHER						AMI-SELF				
			Anger	Happy	Guilt	Excitement	Fear	Recidivism	Anger	Happy	Guilt	Excitement	Fear
*ICU*													
CU traits		.473**											
AMI-OTHER													
Anger	−.137*	−.170**											
Happy	.251**	.123	−.306**										
Guilt	−.200**	−.223**	−.004	−.641**									
Excitement	.153**	.234**	−.324**	.530**	−.763**								
Fear	−.088	−.010	−.078	−.635**	.384**	−.608**							
Recidivism	.093	.232**	−.122	.341**	−.354**	.343**	−.243**						
*AMI*-*SELF*													
Anger	−.078	−.134*	.706**	−.197**	−.015	−.213**	−.093	−.126*					
Happy	.513**	.333**	−.347**	.567**	−.274**	.326**	−.346**	.222**	−.317**				
Guilt	−.455**	−.395**	−.073	−.265**	.557**	−.337**	.043	−.165**	−.225**	−.505**			
Excitement	.505**	.454**	−.346**	.429**	−.464**	.637**	−.363**	.295**	−.341**	.721**	−.619**		
Fear	−.355**	−.174**	.004	−.394**	.113	−.314**	.644**	−.170**	−.144*	−.668**	.203**	−.578**	
Recidivism	.332**	.377**	−.073	.232**	−.274**	.207**	−.099	.222**	−.128*	.419**	−.441**	.460**^.^	−.206**

*ICU* Inventory of Callous–Unemotional traits; *CU traits* Callous–unemotional traits; *YSR* Youth Self-Report; *AMI* Affective Morality Index; *AMI-OTHER* Cognitive Morality Index; *AMI-SELF* Affective Morality Index

* *p* < .05; ** *p* < .01

### Regression Analyses

We performed hierarchical regression analyses to examine the unique and interaction effects of callous–unemotional traits, externalizing problems, and gender on morality judgments. We computed centered variables for ICU and YSR scores by subtracting the total mean score from each individual score and calculated interaction terms by multiplying the centered ICU scores by the centered YSR scores and gender. We controlled for school in our analyses to account for a potential effect of school and country. In the first step of the analyses, we entered dummy variables of the schools as the control variables, in the second step we entered the ICU, YSR, and gender in the model to examine main effects and in the third step we added the interaction terms.

#### Cognitive Morality Judgments

Table 3 presents the results of the regression analyses on cognitive morality judgments. There was no significant main effect of callous–unemotional traits on cognitive morality (Hypothesis 1). However, we found a significant main effect of externalizing problems on excitement, and perceived likelihood of recidivism, suggesting that adolescents with high externalizing problems perceived the protagonist as more excited and more likely to commit a similar act again compared to adolescents with low externalizing problems. Significant interactions between callous–unemotional traits and externalizing problems were not found. There was also a main effect of gender on anger and happiness, indicating that boys perceived the protagonist as feeling happier after committing an antisocial act compared to girls, but girls perceived him as feeling angrier than boys (Hypothesis 3).

**Table 3:** Results of hierarchical regression analyses predicting cognitive morality (AMI-OTHER scores) and recidivism

	*B*	*SE B*	β	*R* ^2^
*Anger*				.064
CU traits	.000	.000	−.020	
Ext. problems	−.001	.001	−.137	
Gender	−.009	.004	−.135*	
CU-Ext. problems	.000	.000	−.011	
CU-Gender	.000	.001	.009	
*Happy*				.119
CU traits	.001	.000	.129	
Ext. problems	.000	.001	−.026	
Gender	.012	.005	.159*	
CU-Ext. problems	.000	.000	.011	
CU-Gender	.001	.001	.098	
*Guilt*				.143
CU traits	−.001	.001	−.090	
Ext. problems	−.001	.001	−.082	
Gender	−.010	.006	−.107	
CU-Ext. problems	.000	.000	−.050	
CU-Gender	−.000	.001	−.021	
*Excitement*				.116
CU traits	.000	.001	−.020	
Ext. problems	.002	.001	.168*	
Gender	.011	.007	.107	
CU-Ext. problems	.000	.000	−.008	
CU-Gender	.001	.001	.081	
*Fear*				.034
CU traits	.000	.001	.025	
Ext. problems	.000	.001	.021	
Gender	−.004	.005	−.055	
CU-Ext. problems	.000	.000	.072	
CU-Gender	−.001	.001	−.183	
*Recidivism*				.183
CU traits	−.032	.060	−.047	
Ext. problems	.219	.077	.193**	
Gender	.721	.613	.070	
CU-Ext. problems	−.004	.008	−.040	
CU-Gender	.053	.090	.053	

*AMI* Affective Morality Index; *AMI-OTHER* Cognitive Morality Index; *AMI-SELF* Affective Morality Index; *CU traits* Callous–unemotional traits; *Ext. problems* Externalizing problems

* *p* < .05; ** *p* < .01

#### Affective Morality Judgments

**Fig. 1:**
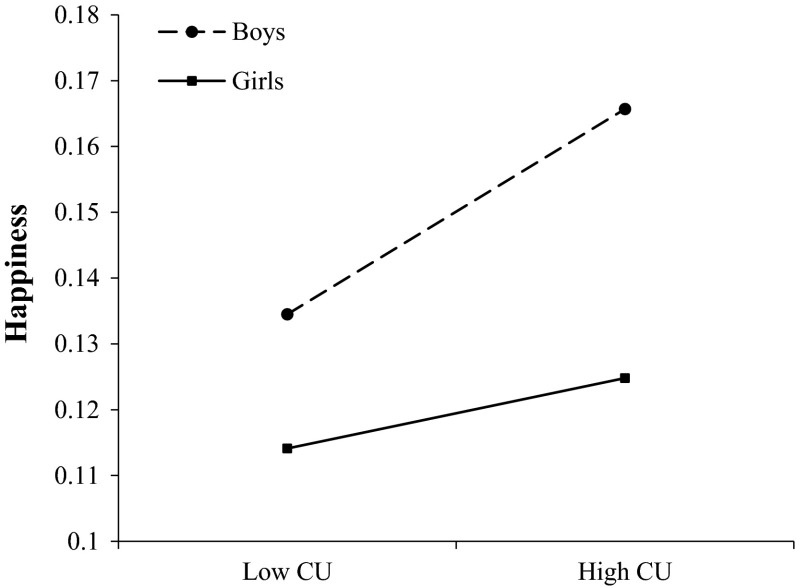
Interaction effects of callous–unemotional (CU) traits (median split) and gender in feelings of happiness when adolescents imagined themselves committing antisocial acts

Table 4 presents the results of the regression analyses on affective morality judgments. There was a significant main effect of callous–unemotional traits on happiness, guilt, excitement, fear, and perceived likelihood of recidivism. Adolescents with high callous–unemotional traits reported increased feelings of happiness and excitement, decreased feelings of guilt and fear, and they estimated an increased likelihood of recidivism when imagining themselves committing similar antisocial acts than adolescents with low callous–unemotional traits (Hypothesis 1). There was also a significant main effect of externalizing problems on happiness, guilt, excitement, and perceived likelihood of recidivism. Adolescents with high externalizing problems reported increased feelings of happiness and excitement, decreased feelings of guilt, and they estimated an increased likelihood of recidivism than adolescents with low externalizing problems (Hypothesis 1). Additionally, a marginally significant (*p* = .056) callous–unemotional traits × externalizing problems interaction on fear was found, indicating that adolescents with high callous–unemotional traits and high externalizing problems exhibited the lowest level of fear when imagining themselves commit similar antisocial acts (Hypothesis 2). Gender also had a significant effect on happiness, guilt, excitement, fear, and perceived likelihood of recidivism. Boys reported increased feelings of happiness and excitement, decreased feelings of guilt and fear, and estimated an increased likelihood of recidivism compared to girls (Hypothesis 3). Finally, there was a significant callous–unemotional traits × gender interaction on happiness, demonstrating that boys with high callous–unemotional traits reported the highest feelings of happiness (see Fig. 1) (Hypothesis 3). There were no significant associations between callous–unemotional traits, externalizing problems, or gender and anger. Overall, callous–unemotional traits, externalizing problems, and gender were strongly associated with affective morality judgments explaining 40.2 % of the variance in happiness, 28.1 % of the variance in guilt, 40.6 % of the variance in excitement, 27 % of the variance in fear, and 45.6 % of the variance in recidivism.

**Table 4:** Results of hierarchical regression analyses predicting affective morality (AMI-SELF scores) and recidivism

	*B*	*SE B*	β	*R* ^*2*^
*Anger*				.045
CU traits	.000	.001	−.033	
Ext. problems	−.001	.001	−.098	
Gender	−.002	.006	−.019	
CU-Ext. problems	.000	.000	.058	
CU-gender	.000	.001	−.011	
*Happy*				.402
CU traits	.001	.000	.239**	
Ext. problems	.001	.000	.172**	
Gender	.024	.004	.320***	
CU-Ext. problems	.000	.000	−.100	
CU-gender	.002	.001	.224**	
*Guilt*				.281
CU traits	−.001	.001	−.237**	
Ext. problems	−.002	.001	−.230**	
Gender	−.016	.005	−.168**	
CU-Ext. problems	.000	.000	−.034	
CU-Gender	−.001	.001	−.073	
*Excitement*				.406
CU traits	.002	.000	.267**	
Ext. problems	.003	.001	.296***	
Gender	.026	.005	.295***	
CU-Ext. problems	.000	.000	−.079	
CU-gender	.001	.001	.063	
*Fear*				.270
CU traits	−.001	.001	−.174*	
Ext. problems	−.001	.001	−.095	
Gender	−.032	.005	−.343***	
CU-Ext. problems	.000	.000	.131	
CU-Gender	−.001	.001	−.153	
*Recidivism*				.456
CU traits	.160	.040	.288***	
Ext. problems	.404	.052	.447***	
Gender	1.518	.418	.182***	
CU-Ext. problems	.003	.005	.031	
CU-gender	−.023	.058	−.028	

*AMI* Affective Morality Index; *AMI-OTHER* Cognitive Morality Index; *AMI-SELF* Affective Morality Index; *CU traits* Callous–unemotional traits; *Ext. problems* Externalizing problems

* *p* < .05; ** *p* < .01; *** *p* < .001

## Discussion

Although moral development and morality judgments in response to moral dilemmas have been consistently examined in relationship to externalizing problems (Malti and Krettenauer 2013; Stams et al. 2006), the association between morality judgments and externalizing problems in response to *antisocial acts* remains obscure. More importantly, it is still unknown whether individuals with externalizing problems and callous–unemotional traits, who are at risk of aggressive and delinquent behavior, experience deviant moral emotions in response to antisocial acts. To our knowledge, only one study investigated this question, indicating that externalizing problems and callous–unemotional traits were related to affective but not cognitive morality (Feilhauer et al. 2013). However, this study was performed in young boys and thus it is unknown whether this effect is also present during adolescence and whether it is specific to males. The aim of this study was to address this question and investigate the role of callous–unemotional traits and externalizing problems in cognitive and affective morality judgments and to elucidate potential gender differences in a community sample of adolescents.

Firstly, cognitive morality was not related to callous–unemotional traits but it was associated with externalizing problems (Hypothesis 1). In particular, adolescents with externalizing problems perceived the protagonist as feeling more excited when committing the antisocial act and more likely to commit a similar antisocial act again compared to adolescents with low externalizing problems. There were no significant interactions between callous–unemotional traits and externalizing problems in cognitive morality. In addition, gender differences were revealed, suggesting that boys perceived the protagonist as feeling happier after committing the antisocial act compared to girls, whereas girls perceived him as angrier than boys (Hypothesis 3). Overall, significant but rather small effect sizes were found for the association between externalizing problems or gender and cognitive morality judgments.

With respect to affective morality, callous–unemotional traits, externalizing problems, and gender were robust and independent predictors explaining a high percentage of the variance in affective morality scores (27 % for fear to 45.6 % for recidivism) (Hypotheses 1, 3). Particularly, callous–unemotional traits and externalizing problems were associated with increased feelings of happiness and excitement, increased likelihood of recidivism, and decreased feelings of guilt when participants imagined themselves committing similar antisocial acts. Contrary to our hypothesis 2, there were no significant interactions between callous–unemotional traits and externalizing problems. There was only a marginally significant interaction on fear, suggesting that adolescents with high callous–unemotional traits and externalizing problems experienced the lowest levels of fear. Moreover, boys reported increased feelings of happiness and excitement, increased recidivism, and decreased feelings of guilt and fear compared to girls (Hypothesis 3). A significant interaction between callous–unemotional traits and gender on happiness scores was found, indicating that boys with high callous–unemotional traits anticipated the highest levels of happiness, whereas girls with low callous–unemotional traits anticipated the lowest levels of happiness when imagining themselves committing an antisocial act.

Taken together, these findings underscore that adolescents with callous–unemotional traits can distinguish between right and wrong and identify the appropriate moral emotions in others according to societal norms, but when they imagine themselves committing antisocial acts, they exhibit deviant moral emotions. Therefore, callous–unemotional traits may be related to deficits in affective morality but not in cognitive morality judgments. These findings are in line with previous studies demonstrating that adults and children with high callous–unemotional traits or psychopathy are capable of understanding the difference between right and wrong as well as the consequences of their actions, but they do not care (Cima et al. 2010; Feilhauer et al. 2013). These deviant moral emotions might reinforce antisocial behavior according to the reward dominance theory, which is highly related to antisocial behavior and callous–unemotional traits (O’Brien and Frick 1996). This theory describes a behavioral pattern that focuses on the immediate positive reward of an action and disregards its potential negative consequences or punishment in the long run (O’Brien and Frick 1996). The feelings of happiness and excitement as well as the lack of guilt and fear could enhance this response style and lead to persistent antisocial behavior. In addition, the association between callous–unemotional traits and affective morality judgments as well as the lack of a relationship with cognitive morality judgments is also highly related to the cumulative evidence on the association between callous–unemotional traits and empathy. Callous–unemotional traits are consistently related to deficits in affective empathy but the results on cognitive empathy are contradictory (Frick et al. 2014). Individuals with callous–unemotional traits are able to understand and recognize the emotional state of others but they have difficulties to respond compassionately and share the others’ emotions. Similarly, they understand the moral emotions that should accompany an antisocial act according to the societal norms but they experience more positive and less negative feelings that might reinforce their antisocial behavior.

Adolescents with externalizing problems expressed deviant moral emotions when imagining themselves committing the antisocial acts, but they also identified higher levels of excitement and higher likelihood of recidivism in the protagonist. Thus, externalizing problems in adolescence may be related to deficits in both cognitive and affective morality judgments. Our results on cognitive morality are inconsistent with the findings by Feilhauer et al. (2013), who found no association between cognitive morality judgments and externalizing problems. This difference is probably due to the distinct age group in our sample. Feilhauer et al. (2013) recruited children at the age of 8–12, whereas we included adolescents. During adolescence, aggressive and delinquent behaviors as well as risk-taking behavior are increased (Crone et al. 2016; Defoe et al. 2015). In addition, risk-taking behavior and sensation seeking have been consistently associated with externalizing problems (Roberti 2004; Swaim et al. 2004; Wilson and Scarpa 2011). It is therefore possible that the higher levels of excitement found in adolescents with high externalizing problems derived from the increased risk-taking and sensation seeking that characterize these individuals. Future research is urged to investigate whether this association is persistent over time or limited to adolescence in order to disentangle the association between cognitive morality and externalizing problems. With respect to affective morality, our findings are in line with the meta-analysis by Malti and Krettenauer (2013) that yielded an association between deviant moral emotions and externalizing problems in response to moral dilemmas. The study by Arsenio et al. (2004) of adolescents with disruptive disorders also found higher levels of happiness in response to antisocial acts. Our study showed deficits in a broad spectrum of moral emotions in affective morality, namely happiness, excitement, guilt, and perception of recidivism. The observed associations between externalizing problems and affective morality judgments suggest that it may be beneficial to target moral emotions for the prevention and treatment of antisocial behavior. It could be useful for prevention programs to investigate the moral emotions associated with antisocial acts in order to identify adolescents with externalizing problems and deviant moral emotions and focus more on how to deal with these moral emotions.

The marginally significant interaction of externalizing problems and callous–unemotional traits on fear when imagining themselves committing similar antisocial acts highlights the role of fear and especially the lack thereof in antisocial behavior. Previous studies have found deficits in passive avoidance learning in individuals with psychopathic traits and externalizing problems, especially in men (Blair et al. 2004; Epstein et al. 2006; Hartung et al. 2002; Newman and Kosson 1986; Thornquist and Zuckerman 1995; Vitale 2011; Vitale et al. 2005). These adolescents tend to focus more on the positive rewarding effects of their antisocial behavior instead of the punishment element or the negative consequences of their behavior. Although in line with these studies, our findings should be interpreted with caution as this interaction did not reach significance. The participants in our study were drawn from the general population and did not exhibit clinical levels of externalizing problems or callous–unemotional traits and thus further research in clinical populations is needed.

Furthermore, our results showed that the effect of callous–unemotional traits and externalizing problems on affective morality was present in both genders, highlighting that these effects are not restricted to males. Additionally, gender was an independent predictor in cognitive and affective morality, suggesting that boys report more deviant moral emotions than girls. These findings are inconsistent with the meta-analysis by Malti and Krettenauer (2013) that did not find gender differences in moral emotions. However, the included studies in this meta-analysis examined moral emotions in response to moral dilemmas and not antisocial acts. Our study focused specifically on antisocial acts and the revealed gender differences highlight that, even though both males and females understand whether an action is considered moral or not, boys tend to attribute more positive and less negative emotions to antisocial acts. Extensive previous research has shown that adolescent boys exhibit higher levels of externalizing problems, callous–unemotional traits, and risk-taking behavior than girls (Archer 2004; Bongers et al. 2004; Broidy et al. 2003; Byrnes et al. 1999; Chun and Mobley 2010; Cook et al. 2015; Essau et al. 2006; Euler et al. 2015; Meier et al. 2008; Nichols et al. 2006; Stams et al. 2006; Urben et al. 2015). Adolescent boys present more sensitivity to high reward and insensitivity to punishment than girls that is suggestive of a reward dominance response style (Grose-Fifer et al. 2014). We argue that the link between male gender and aggression might be influenced by a combination of deviant moral emotions and a predominant reward dominance response style. Specifically, we propose that boys who exhibit aggressive behavior might follow a path from deviant moral emotions and reward dominance response style to aggressive and delinquent behavior. Relatedly, girls’ ability to identify and anticipate the appropriate moral emotions might be a protective factor against antisocial behavior that needs further exploration. Girls exhibit lower levels of risk-taking behavior and increased levels of empathy compared to boys in adolescence and adulthood (Eisenberg and Lennon 1983; Gullone and Moore 2000; O’Brien et al. 2013; Thompson and Voyer 2014). The ability to share the emotions of others might be linked to moral emotions attributed to antisocial acts and thus prevent girls from engaging in antisocial behavior. The relationship between empathy and moral emotions should be further examined in future longitudinal studies to better understand whether and how they interact with each other in the course of development from childhood to adolescence and their association with antisocial behavior.

Our findings are also relevant to the treatment of antisocial behavior and the criminal justice system. High callous–unemotional traits and psychopathy are predictive of severe antisocial behavior, criminal activity, incarceration, and recidivism (Frick et al. 2014; Frick and White 2008). Thus, there is a great need of effective interventions specifically tailored to callous–unemotional traits and psychopathy. Several emotional and empathy training programs have been developed aiming to improve emotion recognition and cognitive/affective empathy for diverse psychiatric disorders, such as autism spectrum, disorders, schizophrenia, depression, and conduct problems (Dadds et al. 2012; Datyner et al. 2016; Kimber et al. 2008a, b; Klimecki et al. 2014; Pecukonis 1990). A few of these programs have been applied to children and adolescents with externalizing problems and callous–unemotional traits and have presented a positive effect on affective empathy (Dadds et al. 2012; Datyner et al. 2016; Pecukonis 1990). We propose that it could be highly beneficial to combine affective empathy training with affective morality in order to help individuals with callous–unemotional traits share and respond compassionately to the emotions of others and learn to express more appropriate moral emotions related to criminal acts. For instance, a person who committed an aggressive act toward someone else would learn not only to acknowledge but also to feel the pain of the person they hurt, and the morality component would help them to express more appropriate moral emotions, such as more negative (guilt) and less positive (happiness) feelings for the criminal act.

Finally, in relation to the criminal justice system, our findings have implications for restorative justice. Restorative justice is a process that includes both the offender and the victim in an effort to initiate a dialogue that will lead to feelings of empathy and remorse in the offender. Then the offender takes responsibility for their actions and eventually helps the victim feel a sense of justice and empowerment (Gavrielides and Worth 2013). Empathy and affective morality are core components of restorative justice and thus the lack thereof condemn the process (Koufouli and Tollenaar 2016). Consequently, individuals with callous–unemotional traits that have impairments in affective empathy and morality judgments might not be the best candidates for restorative justice and these characteristics should be taken into consideration before proceeding to this process. Alternatively, it could be beneficial for individuals with callous–unemotional traits or psychopathy to follow interventions that aim to improve empathic responding and affective morality and then participate in restorative justice. In addition, the association between externalizing problems and/or callous–unemotional traits and recidivism has important implications, as the primary aim of the justice system is to reduce recidivism. Callous–unemotional traits and externalizing problems are predictive of recidivism in adolescents (Asscher et al. 2011; Cottle et al. 2001). Recidivism is also linked to lower stages of moral development and moral emotions (empathy, shame, guilt) (Van Vugt et al., 2011). Our results are in line with these findings as they showed that adolescents with callous–unemotional traits or externalizing problems estimated an increased likelihood of recidivism and recidivism was correlated with deviant moral emotions. Thus, our study further supports the idea that targeting moral emotions during treatment might be useful in the prevention of recidivism.

Several limitations of this study should also be mentioned to take a better perspective of the generalizability of our results. Firstly, the study included a community sample of adolescents and thus further exploration is needed to establish whether the same emotional processes and moral judgments can be found in clinical or incarcerated populations. A number of adolescents who were approached did not participate in the study due to lack of interest or absence from the school, which might be related to externalizing problems and delinquency and thus they might also be characterized by different moral emotions. Additionally, although the gender differences were robust, it is noteworthy that the AMI includes vignettes with male protagonists and thus it is unclear whether this might have played a role in the elicited moral emotions. It is possible that adolescent girls would have a different perspective for the moral emotions of a female protagonist. Relatedly, the antisocial acts described in the vignettes are characteristic of acts committed by boys and consequently they might not elicit strong moral emotions for girls when imagining themselves committing similar acts. The addition of vignettes with female protagonists would significantly improve the validity of the instrument and allow us to draw more solid conclusions for both genders.

## Conclusion

Overall, the present study revealed four important findings: (a) callous–unemotional traits were strongly related to deficits in affective but not in cognitive morality judgments, (b) externalizing problems were associated with deficits in both affective and cognitive morality judgments although the association with affective morality was notably stronger, (c) a similar pattern of strong deficits in affective morality and a weaker relationship with cognitive morality was found in boys, and (d) boys with high callous–unemotional traits exhibited the highest levels of happiness in affective morality. Although moral development has been consistently associated with antisocial behavior in response to moral dilemmas, research on a broad spectrum of moral emotions in response to antisocial acts is still limited. Our study filled this gap and revealed that adolescents with high callous–unemotional traits can identify the appropriate moral emotions in others but they anticipate higher positive (happiness, excitement) and lower negative (guilt, fear) emotions when imagining themselves committing antisocial acts. In contrast, adolescents with externalizing problems and boys reported deviant moral emotions in others as well as themselves. This difference emphasizes the distinctive nature of callous–unemotional traits and the need of more tailored interventions. Overall, the present study contributes substantially to our knowledge about the underlying mechanisms and moral emotions associated with antisocial and delinquent behavior with crucial implications for theory and clinical practice as well as the criminal justice system.
